# Cervical cancer subtypes harbouring integrated and/or episomal HPV16 portray distinct molecular phenotypes based on transcriptome profiling of mRNAs and miRNAs

**DOI:** 10.1038/s41420-019-0154-x

**Published:** 2019-03-25

**Authors:** Paramita Mandal, Sweta Sharma Saha, Shrinka Sen, Amrapali Bhattacharya, Nitai P. Bhattacharya, Sudha Bucha, Mithun Sinha, Rahul Roy Chowdhury, Nidhu Ranjan Mondal, Biman Chakravarty, Tanmay Chatterjee, Sudipta Roy, Ansuman Chattapadhyay, Sharmila Sengupta

**Affiliations:** 1 National Institute of Biomedical Genomics, Kalyani, West Bengal India; 2 Crystallography & Molecular Biology Division, Saha Institute of Nuclear Physics, 1/AF Bidhannagar, Kolkata, 700064 India; 3 Department of Gynecology, Saroj Gupta Cancer Centre and Research Institute, Kolkata, India; 4 Sri Aurobindo Seva Kendra, 1H, Gariahat Road (S) Jodhpur Park, Kolkata, 700068 West Bengal India; 5 Health Science Library System, University of Pittsburgh, Pittsburgh, PA USA; 6 Present Address: Department of Zoology, The University of Burdwan, Burdwan, West Bengal India; 7 Present Address: Section of Haematology/Oncology, Department of Medicine, university of Chicago, 5841 S Maryland Ave MC 2115, Chicago, IL 60637 USA; 8 Present Address: Molecular Biology and Genetics Unit, Jawaharlal Nehru Centre for Advanced Scientific Research, Jakkur, Bengaluru, Karnataka 560064 India; 9 Present Address: Comprehensive Wound Center, Center for Regenerative Medicine and Cell Based Therapies, The Ohio State University, Columbus, OH USA

## Abstract

Heterogeneity in cervical cancers (CaCx) in terms of HPV16 physical status prompted us to investigate the mRNA and miRNA signatures among the different categories of CaCx samples. We performed microarray-based mRNA expression profiling and quantitative real-time PCR-based expression analysis of some prioritised miRNAs implicated in cancer-related pathways among various categories of cervical samples. Such samples included HPV16-positive CaCx cases that harboured either purely integrated HPV16 genomes (integrated) and those that harboured episomal viral genomes, either pure or concomitant with integrated viral genomes (episomal), which were compared with normal cervical samples that were either HPV negative or positive for HPV16. The mRNA expression profile differed characteristically between integrated and episomal CaCx cases for enriched biological pathways. miRNA expression profiles also differed among CaCx cases compared with controls (upregulation—miR-21, miR-16, miR-205, miR-323; downregulation—miR-143, miR-196b, miR-203, miR-34a; progressive upregulation—miR-21 and progressive downregulation—miR-143, miR-34a, miR-196b and miR-203) in the order of HPV-negative controls, HPV16-positive non-malignant samples and HPV16-positive CaCx cases. miR-200a was upregulated in HPV16-positive cervical tissues irrespective of histopathological status. Expression of majority of the predicted target genes was negatively correlated with their corresponding miRNAs, irrespective of the CaCx subtypes. E7 mRNA expression correlated positively with miR-323 expression among episomal cases and miR-203, among integrated cases. miR-181c expression was downregulated only among the episomal CaCx cases and negatively correlated with protein coding transcript of the proliferative target gene, *CKS1B* of the significantly enriched “G2/M DNA Damage Checkpoint Regulation” pathway among CaCx cases. Thus, the two CaCx subtypes are distinct entities at the molecular level, which could be differentially targeted for therapy. In fact, availability of a small molecule inhibitor of *CKS1B*, suggests that drugging *CKS1B* could be a potential avenue of treating the large majority of CaCx cases harbouring episomal HPV16.

## Introduction

Cervical cancer (CaCx) appears to be the most common malignancy in Indian women, characterised by high incidence and mortality rates that are attributable to late detection and lack of access to affordable health care. Oncogenic human papillomavirus (HPV) infections are the major aetiological factors, with a predominance of HPV16 that accounts for over 50% of such cancers^1^. Viral oncoprotein E6 is known to interact with the tumour suppressor *p53* and E7, with the PDZ domain of cellular proteins and *Rb*, thereby facilitating neoplastic progression through loss of cellular homoeostasis^2^. Loss of E2 repressor activity, as a consequence of integration of the virus into the host genome, is known to impart proliferative advantage to the host cells^3^. Besides this, our earlier studies have highlighted mechanisms that act to block the E2-mediated repression of the P97 promoter^4^ in CaCx cases harbouring episomal HPV16 and revealed that such cancers harbour higher viral load and E7 expression in contrast to the ones that portray integrated HPV16.

Cervical carcinogenesis, under the impact of oncogenic HPV infections, could be influenced by several epigenetic mechanisms like DNA methylation, histone modifications, by microRNAs, etc., through perturbations of gene expression profiles. In this communication, we chose to focus on the expression of host mRNAs and miRNAs in various categories of cervical samples spanning the discrete stages of CaCx development. These small non-coding RNAs, through recognition of sequence-complementary target elements, can either translationally suppress or catalytically degrade both cellular and viral RNAs^5,6^. Both DNA and RNA viruses have evolved mechanisms to degrade, boost, or hijack cellular miRNAs to benefit the viral life cycle^7^. Therefore, a glimpse of the deregulated expression of mRNAs and miRNAs in CaCx cases might offer some insights into the mechanisms of CaCx pathogenesis among the two subtypes that bear integrated HPV16 and episomal HPV16 in presence or absence of integration, subsequently referred to as integrated and episomal CaCx cases, respectively.

About 50% of the known human miRNAs^8^ are located at cancer-associated regions^9^ of the genome and a number of miRNAs are located close to HPV integration sites. Host miRNAs are also capable of influencing viral life cycles, viral tropism, and the pathogenesis of viral diseases^5^. Therefore, miRNA-mediated epigenetic regulation of gene expression might play a role in virus associated cancers, like CaCx.

Some studies have strongly suggested that miRNA profiling is more robust than mRNA profiling in determining the heterogeneity among cancers^10^. The studies on CaCx^11,12^ have also demonstrated the association of miRNA deregulation with tumour heterogeneity. The heterogeneity among CaCx in terms of physical status of HPV16 genomes by our group^13^, prompted us to explore the mRNA and miRNA signatures among the different categories of CaCx samples.

Hence, we tested the hypothesis that CaCx cases with integrated and episomal HPV16 genomes differ with respect to such gene expression profiles and corresponding enriched pathways. We further explored whether a prioritised set of miRNAs, implicated in various cancer-related pathways, show altered expression and play a role in CaCx pathogenesis under the impact of HPV16 infection. Subsequently, we determined whether the miRNAs showing altered expression in CaCx cases, correlate with (i) the physical status of HPV16 genomes (episomal and integrated), (ii) expression of the oncoprotein E7, (iii) the expression of cellular target genes of specific biological pathways and checked the relationship, if any, between such target genes and viral oncoprotein E7.

## Results

### Microarray-based gene expression profiling reveals characteristic differences among CaCx cases harbouring episomal and integrated HPV16 genomes

To identify the differences between CaCx cases harbouring episomal and integrated HPV16 genomes, we first attempted to identify global gene expression level changes between the two categories of CaCx cases. Our microarray-based analysis revealed that 334 genes were differentially upregulated and 253 were differentially downregulated among episomal CaCx cases, whereas 1373 genes were differentially upregulated and 1240 were differentially downregulated among integrated CaCx cases, compared with the HPV-negative control samples (Figure S1). Ingenuity pathway analysis (IPA) revealed that biological processes such as cell proliferation, retroviridae infection, and viral Infections were the most significantly enriched among the episomal CaCx cases. On the other hand, biological processes such as transcription, proliferation of tumour cells, and protein metabolism were most significantly enriched among integrated CaCx cases (Table S1). IPA also revealed that role of CTLA4 signalling in cytotoxic T lymphocytes and T-cell receptor signalling were the top significantly altered pathways among the episomal CaCx cases, whereas EIF2 signalling, regulation of eIF4 and p70S6K signalling, mitochondrial dysfunction, and mTOR signalling were the top significantly altered pathways among integrated CaCx cases (Table S2). Moreover, the upstream regulators involved in alteration of gene expression, differed distinctly among the two CaCx subtypes (Table S3). Such observations further strengthen our hypothesis that the episomal and integrated CaCx cases represent two distinct molecular subtypes.

### miRNAs reveal altered expression among CaCx cases compared with HPV-negative controls and HPV16-positive non-malignant samples

**Fig. 1:**
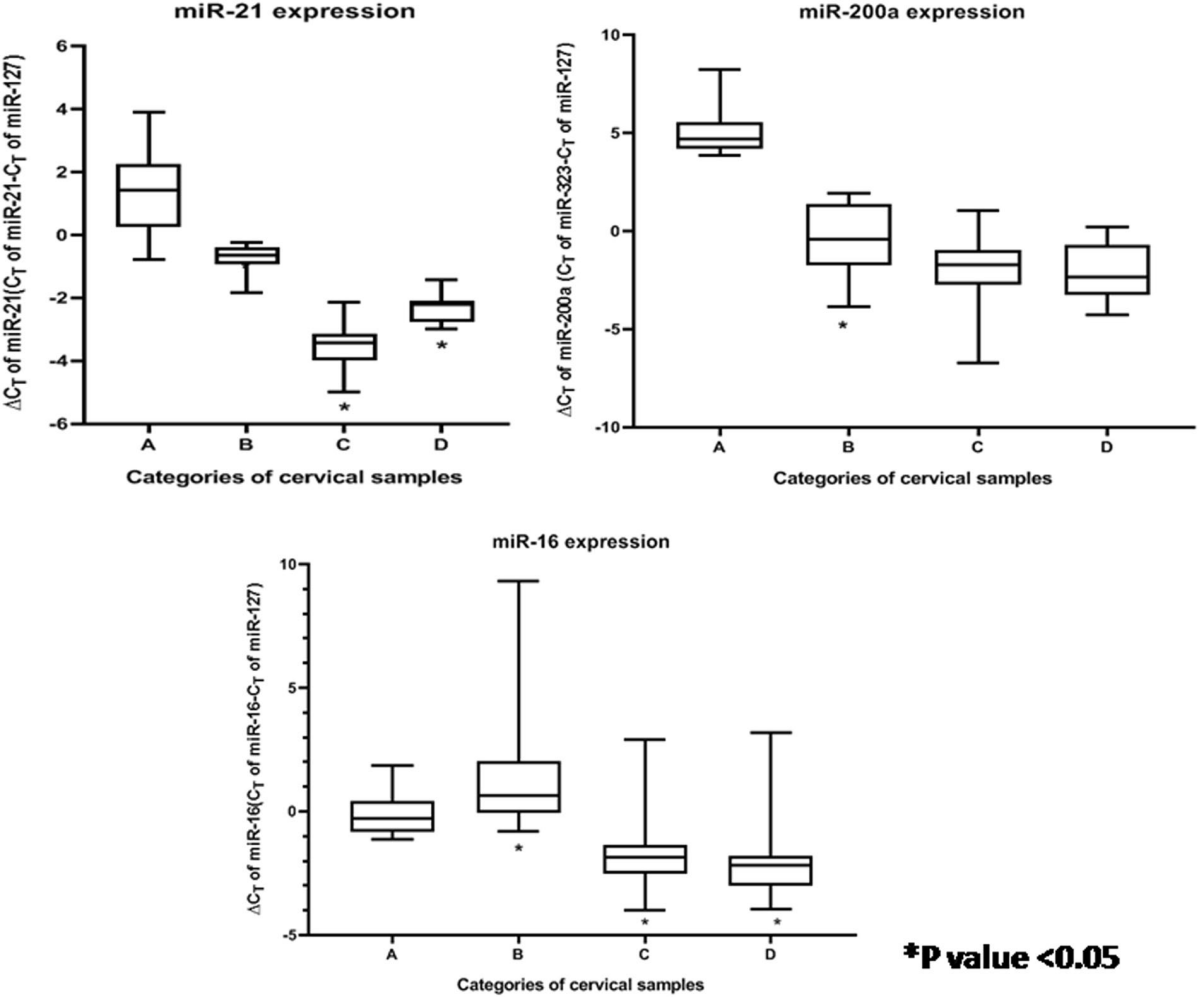
Box plots representing distribution of upregulated miRNA expressions (miR-21, miR-200a, and miR-16, normalised with endogenous control RNU6b and miR-127) among different categories of cervical samples. Lower ∆CT means higher expression. A = HPV-negative control samples (*n* = 25), B = HPV16-positive non-malignant samples (*n* = 25), C = HPV16-positive episomal CaCx cases (*n* = 43), D = HPV16-positive integrated CaCx cases (*n* = 19)

**Fig. 2:**
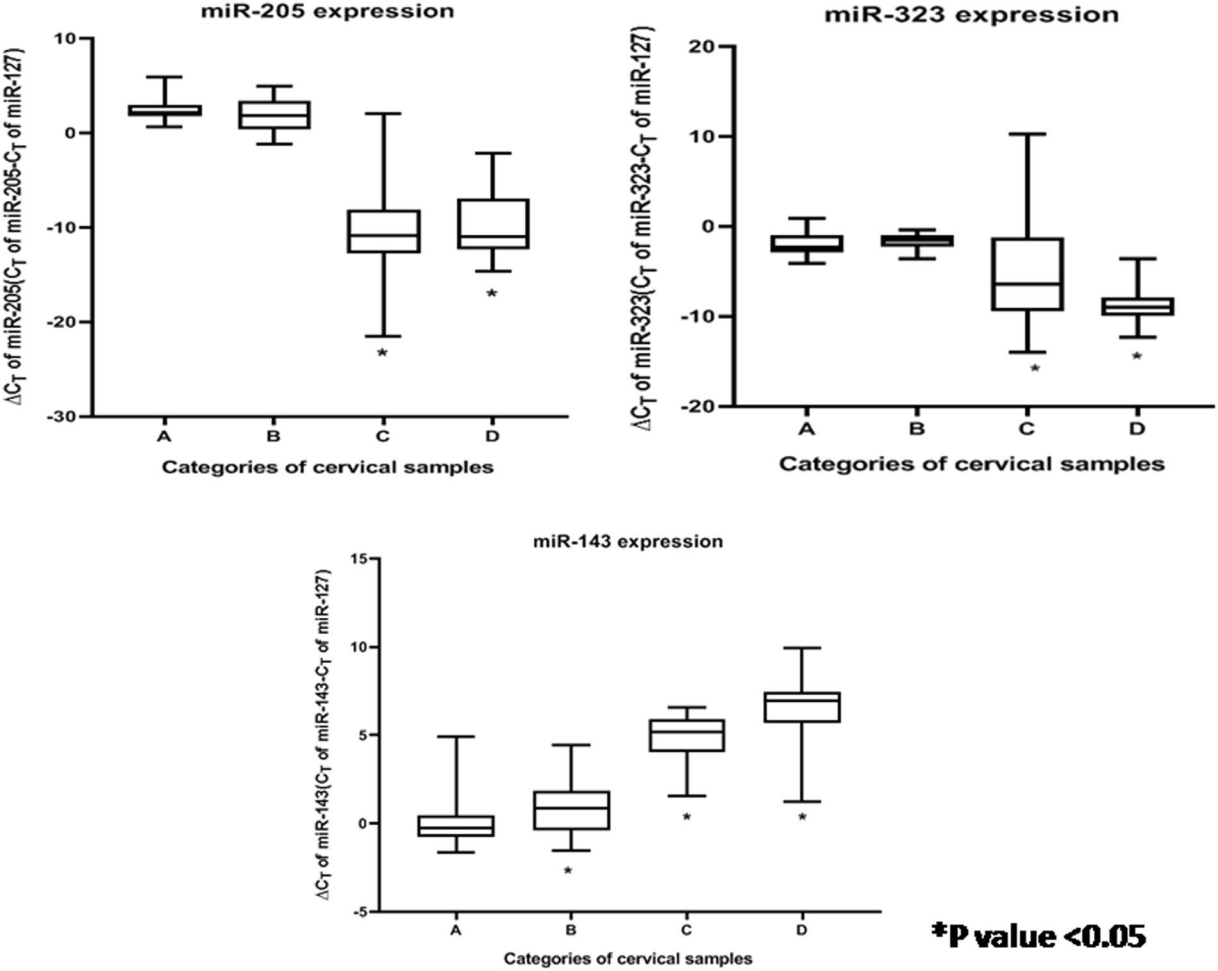
Box plots representing distribution of upregulated and downregulated miRNA expressions (miR-205, miR-323, and miR-143, normalised with endogenous control RNU6b and miR-127) among different categories of cervical samples. Lower ∆CT means higher expression. A = HPV-negative control samples (*n* = 25), B = HPV16-positive non-malignant samples (*n* = 25), C = HPV16-positive episomal CaCx cases (*n* = 43), D = HPV16-positive integrated CaCx cases (*n* = 19)

**Fig. 3:**
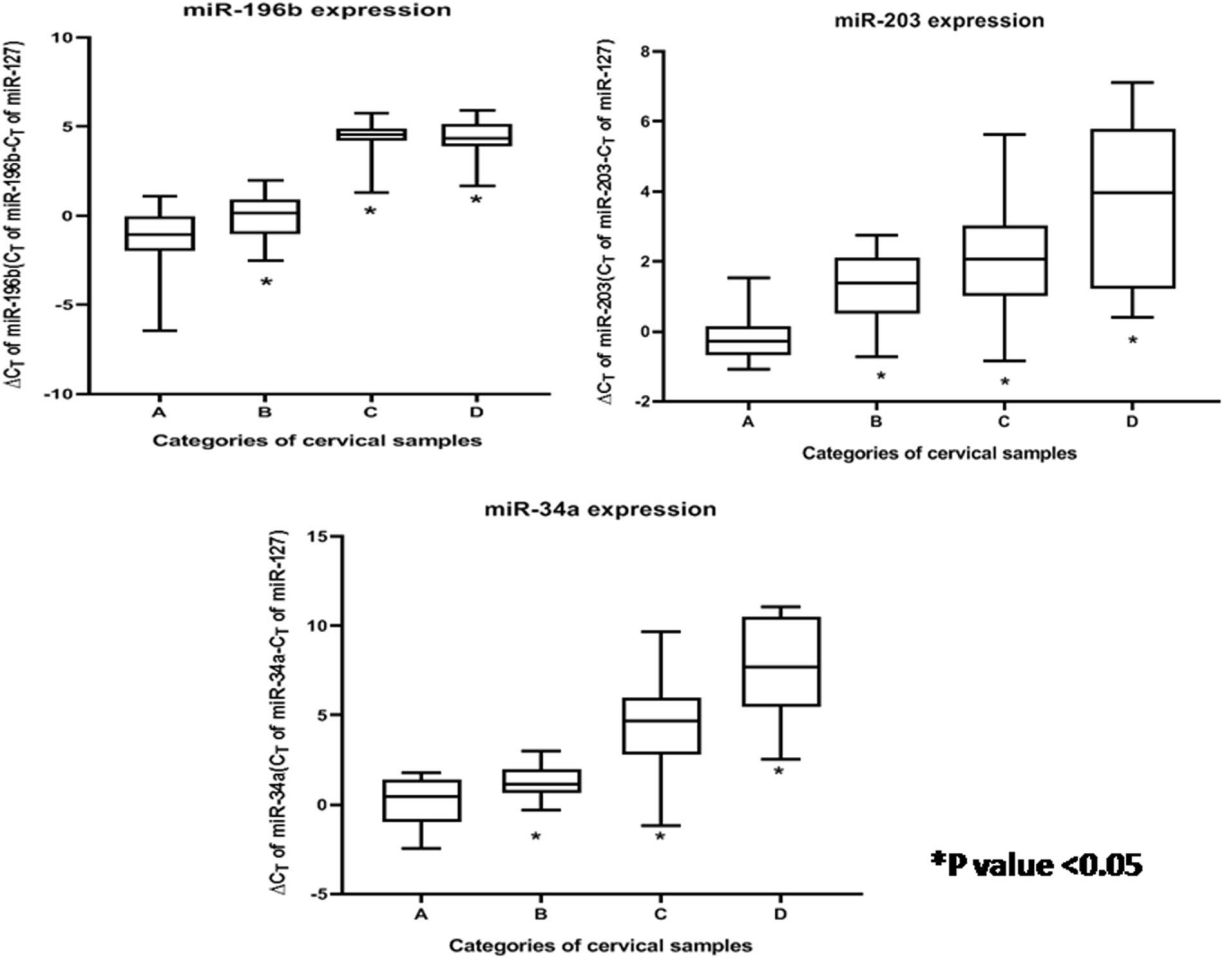
Box plots representing distribution of downregulated miRNA expressions (miR-34a, miR-196b, and miR-203, normalised with endogenous control RNU6b and miR-127) among different categories of cervical samples. Lower ∆CT means higher expression. A = HPV-negative control samples (*n* = 25), B = HPV16-positive non-malignant samples (*n* = 25), C = HPV16-positive episomal CaCx cases (*n* = 43), D = HPV16-positive integrated CaCx cases (*n* = 19)

The 34 miRNAs selected for this study belonged to eight different categories by function, as depicted in Table S4. Of these, nine miRNAs revealed significant alterations in expression (*p* value < 0.05) among CaCx cases compared with HPV-negative controls, as well as HPV16-positive non-malignant samples as depicted in Table 1. Among these, four miRNAs (miR-21, miR-16, miR-205, and miR-323) were significantly overexpressed, while significant decreased expression was recorded in case of four miRNAs (miR-143, miR-196b, miR-203, and miR-34a). miR-200a, however, showed a statistically significant increased expression by 37.53-fold, among CaCx cases compared with HPV-negative controls but not when compared with HPV16-positive non-malignant samples. The expression status of all the altered miRNAs are depicted in Table 1, Figure S2, Fig. 1, Fig. 2 and Fig. 3.

**Table 1:** Expression status of different categories of miRNAs among CaCx cases compared with HPV-negative controls and HPV16-positive non-malignant samples

Category	microRNAs	Comparisons							
		Episomal CaCx cases vs HPV-negative controls		Integrated CaCx cases vs HPV-negative controls		Episomal CaCx cases vs HPV16-positive non-malignant samples		Integrated CaCx cases vs HPV16-positive non-malignant samples	
		Mann–Whitney *U* *p* value (fold change)	FDR of 0.05	Mann–Whitney *U* *p* value (fold change)	FDR of 0.05	Mann–Whitney *U* *p* value (fold change)	FDR of 0.05	Mann–Whitney *U* *p* value (fold change)	FDR of 0.05
Enhance tumour growth and proliferation	miR-21	**<0.001** (25.46); upregulation	0.0015	<0.001 (15.01); upregulation	0.0015	**<0.001** (6.54); upregulation	0.0015	**<0.001** (3.86); upregulation	0.0015
	miR-200a	**<0.001** (48.5); upregulation	0.01	<0.001 (33.93); upregulation	0.01	0.172 (5.11)	0.02	0.062 (2.536)	0.02
Differentiation regulatory miRNAs	miR-143	**<0.001** (−41.07); downregulation	0.003	<0.001 (−76.24); downregulation	0.003	**<0.001** (−13.36); downregulation	0.003	**<0.001** (−24.08); downregulation	0.003
Target tumour suppressors and tumour suppressor miRNAs	miR-16	**<0.001** (3.58); upregulation	0.006	<0.001 (4.32); upregulation	0.006	**<0.001** (5.96); upregulation	0.006	**<0.001** (15.78); upregulation	0.006
	miR-205	**<0.001** (116.07); upregulation	0.012	<0.001 (156.9); upregulation	0.012	**<0.001** (94.18); upregulation	0.01	**<0.001** (124.56); upregulation	0.01
	miR-323	**0.003** (9.87); upregulation	0.014	0.006 (15.77); upregulation	0.014	**<0.001** (37.6); upregulation	0.012	**<0.001** (58.23); upregulation	0.012
	miR-34a	**<0.001** (−28.6); downregulation	0.009	<0.001 (−20); downregulation	0.009	**<0.001** (−15.09); downregulation	0.009	**<0.001** (−9.33); downregulation	0.009
Metastatic suppressor miRNAs	miR-196b	**<0.001** (−39.34); downregulation	0.004	<0.001 (−47.21); downregulation	0.004	**<0.001** (−12.65); downregulation	0.004	**<0.001** (−20.88); downregulation	0.004
Mesenchymal to epithelial transition miRNAs	miR-203	**<0.001** (−4.77); downregulation	0.007	<0.001 (−5.79); downregulation	0.007	**<0.001** (−1.33); downregulation	0.007	**<0.001** (−1.76); downregulation	0.007
Metastatic miRNAs	None	None	None	None	None	None	None	None	None

Bold indicates significant *p* value

Likewise, seven miRNAs revealed significant alterations in expression among HPV16-positive non-malignant samples, compared with HPV-negative controls. Of these, two miRNAs (miR-21, miR-200a) revealed significant increased expression and five miRNAs (miR-143, miR-16, miR-196b, miR-203, and miR-34a) showed significantly decreased expression. The expression status of all such altered miRNAs among the HPV16-positive non-malignant sample group is depicted in Table S5, Figure S2, Fig. 1, Fig. 2, and Fig. 3.

Progressive increase of miR-21 expression and progressively decreased expression of miR-143, miR-34a, miR-196b, and miR-203 was recorded with increase in severity of cervical status through HPV-negative control samples, HPV16-positive non-malignant samples and CaCx cases. Such trends were found to be statistically significant (*p* < 0.001). On the contrary, miR-16 revealed a deviation of the trend with decreased expression among HPV16-positive non-malignant samples, followed by increased expression among CaCx cases compared with HPV-negative controls, as depicted in Figure S3. Such observations are suggestive of the fact that miRNA deregulations could potentially serve as risk markers of CaCx development.

### Lack of profound impact of physical status of HPV16 genome (episomal and integrated) on expression of majority of miRNAs in CaCx cases, excepting for miR-181c

**Fig. 4:**
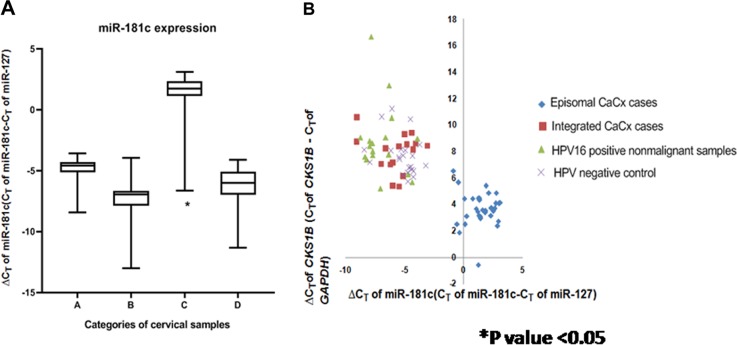
Expression status of miR-181c and its correlation with CKS1B expression. **a** Box plots representing distribution of miR-181c expression among different categories of cervical samples. **b** Linear regression analysis of the correlation between *CKS1B* mRNA expression and miR-181c expression in all cervical samples (*p* = 0.03) Lower ∆C_*T*_ means higher expression. A = HPV-negative control samples (*n* = 25), B = HPV16-positive non-malignant samples (*n* = 25), C = HPV16-positive episomal CaCx cases (*n* = 43), D = HPV16-positive integrated CaCx cases (*n* = 19)

In view of the biological relevance of episomal HPV16 among CaCx cases^13^, expression data of all 34 miRNAs considered in this study was further analysed to determine whether they showed differentially altered expression among the CaCx cases with episomal and integrated HPV16, when compared with HPV-negative controls and HPV16-positive non-malignant samples. The sole miRNA that revealed significantly altered expression only among episomal CaCx cases was miR-181c, as depicted in Fig. 4. miR-181c expression was significantly increased in such cases (fold change = −2.34-fold, *p* < 0.001), compared with both HPV-negative and HPV16-positive control samples (fold change = −15.89 fold; *p* < 0.001). Similar analysis considering CaCx cases with integrated HPV16, failed to show differential expression of miR-181c among such CaCx cases. Thus, HPV16 physical status within CaCx cases failed to portray any profound effect on majority of the miRNAs as depicted in Table 1.

### HPV16 E7 mRNA expression levels activate expression of miR-323 resulting in upregulation among CaCx cases

**Fig. 5:**
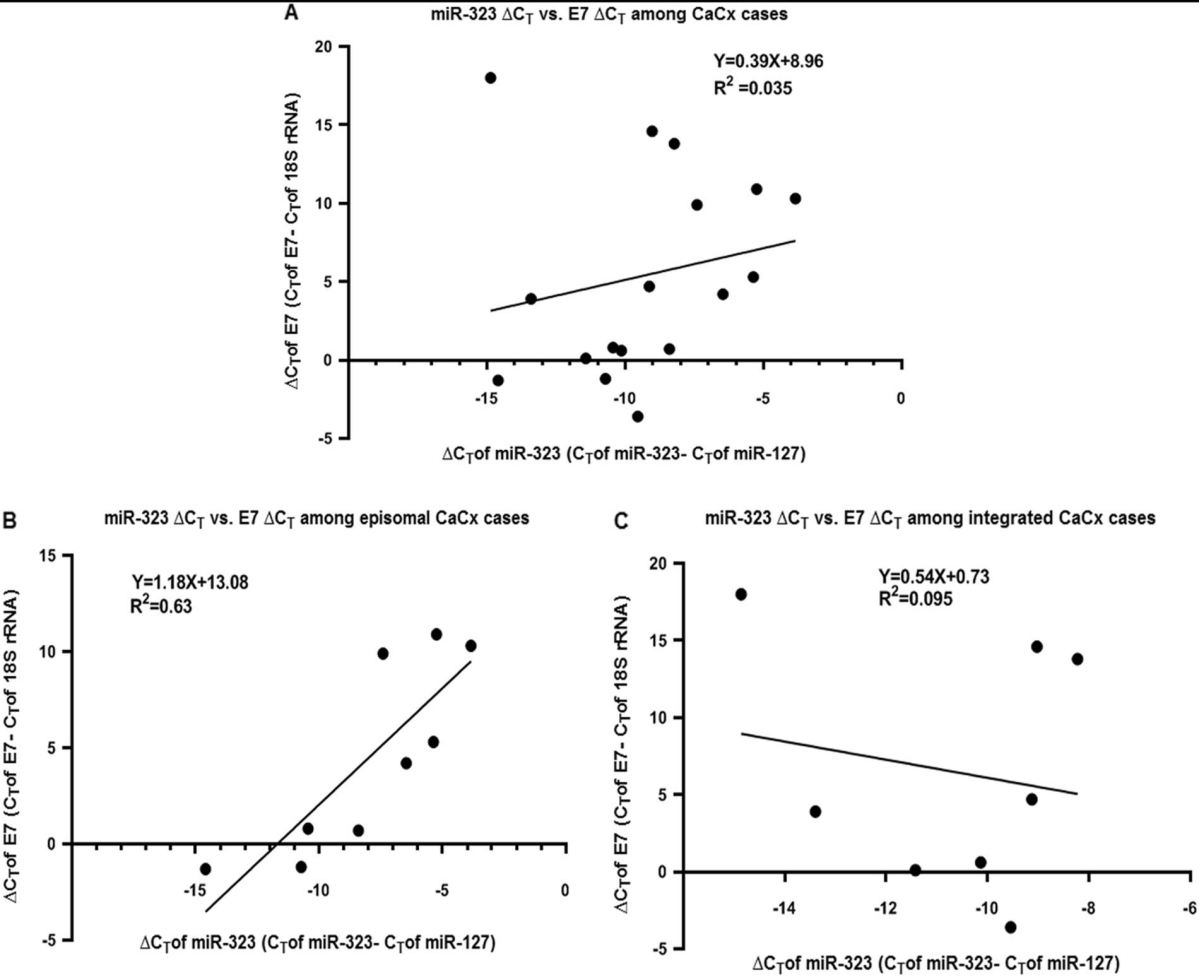
Correlation between miR-323 and E7 expression among cervical cancers. Linear regression analysis of the correlation between miR-323 expression and E7 mRNA expression in (**a**) CaCx cases (episomal and integrated; *p* = 0.471), (**b**) episomal CaCx cases (*p* = 0.011), and (**c**) integrated CaCx cases (*p* = 0.711). Lower ∆C_T_ means higher expression

Based on our clinical sample data we identified that only two miRNAs (miR-203 and miR-323) were significantly and positively correlated with E7 mRNA expression, among the CaCx cases. Interestingly, the correlation between E7 and miR-323 (Fig. 5) was obvious among CaCx cases harbouring episomal HPV16, whereas the correlation between E7 and miR-203 (Figure S4) was evident among CaCx cases with integrated HPV16. As the correlation between miR-203 and E7 has already been documented in earlier studies^14^, we used an in vitro approach to confirm the correlation between miR-323 expression and E7 expression in CaCx cases.

**Fig. 6:**
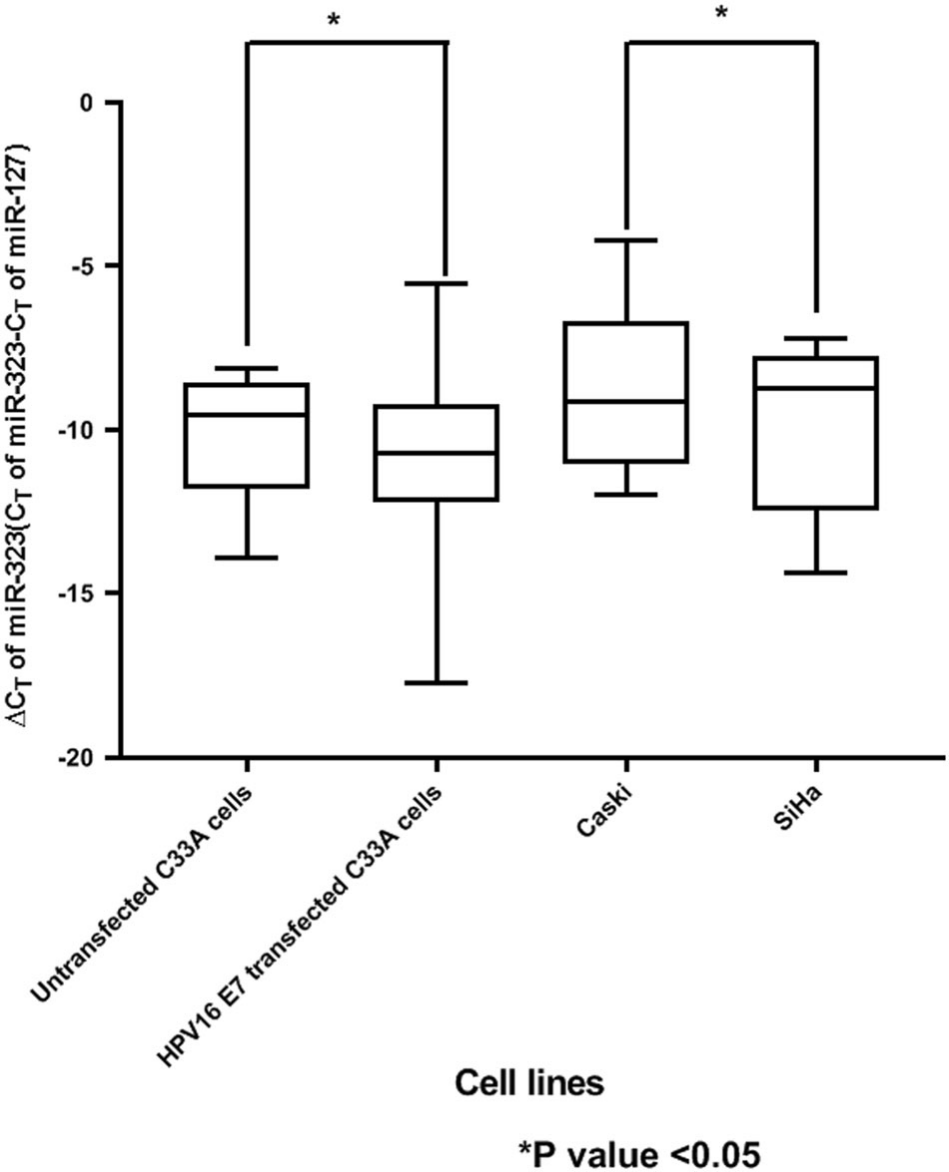
miR-323 expression among various categories of CaCx cell lines. Lower ∆C_T_ means higher expression

We transfected the HPV-negative CaCx cell line, C33A with HPV16 E7 cloned into mammalian expression vector pcDNA3.1 (+), as explained in our previous study^15^. This resulted in a significant increase in miR-323 expression (8.17-fold; *p* = 0.032) concomitant with HPV16 E7 expression (Fig. 6) in such cells. We also determined miR-323 expression in HPV16-positive CaCx cell lines, SiHa, and Caski. Expression of miR-323 was found to be higher among SiHa cells (2.82-fold; *p* = 0.016) that portray relatively higher E7 expression^15^, as compared WITH Caski cells (Fig. 6). Thus, both the observations confirmed that E7 could probably activate miR-323 expression in CaCx cases.

### Target genes of majority of miRNAs that differ in expression between CaCx cases harbouring episomal and integrated HPV16, also portrayed similar expression level difference between such cases

miRNAs are known to target multiple transcripts regulating important cellular processes such as cell proliferation, apoptosis, cellular metastasis, and many more^16^. To check the impact of deregulated expression of the nine significantly altered miRNAs including miR-181c, on their target genes, we identified the expression profiles of the predicted targets from the microarray-based gene expression analysis (Table S6). We selected the target genes of the above mentioned 10 miRNAs, considering only those targets that portrayed ≥ or ≤ 2 fold change in expression as compared with the controls. Majority of such target genes of the corresponding miRNAs seemed to be experimentally non-validated, as per the databases considered for target identification as detailed under the “Methods” section. For miR-181c, only the expression status of target genes in case of episomal CaCx cases were considered for expression changes, as this miRNA was differentially expressed, compared with controls, only among the episomal cases but remained unaltered in expression among the HPV16-integrated CaCx cases.

### Overexpression of miR-181c target gene, CKS1B, among episomal CaCx cases

According to microarray-based gene expression analysis, 44 miR-181c target genes showed differential expression at more than equal to twofolds among the episomal CaCx cases. These genes were also found to be overexpressed among the episomal CaCx cases compared with integrated CaCx cases (Table S6). Out of these 44 differentially overexpressed genes, *CKS1B* was the only gene that was captured in a significantly enriched pathway among CaCx cases, upon employing IPA (Table S2).

The microarray-based gene expression profile result of *CKS1B* is depicted in Table S7. Real-time quantitative PCR-based validation of the expression of *CKS1B* on a larger set of cervical samples, revealed differentially increased expression of *CKS1B* by 21.16-fold (*p* < 0.001) among CaCx cases (episomal HPV16; *n* = 43) compared with HPV16-positive non-malignant samples (*n* = 25) and by 18.5-fold (*p* < 0.001) in comparison with HPV-negative controls (*n* = 25), as depicted in Figure S5. CaCx cases with integrated HPV16 failed to show significant alteration of the expression of this gene, compared with HPV16-positive non-malignant samples and HPV-negative controls.

The expression of *CKS1B* was inversely and significantly correlated with miR-181c expression (*p* = 0.03) based on pair-wise correlation analysis of various categories of cervical samples, as depicted in Fig. 4. The episomal CaCx cases appeared to cluster separately, whereas the integrated CaCx cases clustered together with HPV16-positive non-malignant samples and HPV-negative controls. Such analysis further confirmed that *CKS1B* was differentially overexpressed among episomal CaCx cases possibly as a consequence of differentially decreased expression of miR-181c among such cases. On the other hand, *CKS1B* is transcriptionally regulated by *MYCN*, which has been predicted to be inhibited among the CaCx cases harbouring integrated HPV16, based on IPA in this study (Table S2). This observation was therefore, in concordance with the fact that *CKS1B* failed to show any alteration in expression, compared with controls, in such CaCx subtypes (Table S5). Such observations also reflect that the two CaCx subtypes with episomal and integrated HPV16 are distinct, molecularly.

### miR-181c target gene, CKS1B, correlates negatively with HPV16 E7 expression among the HPV16-integrated CaCx cases

**Fig. 7:**
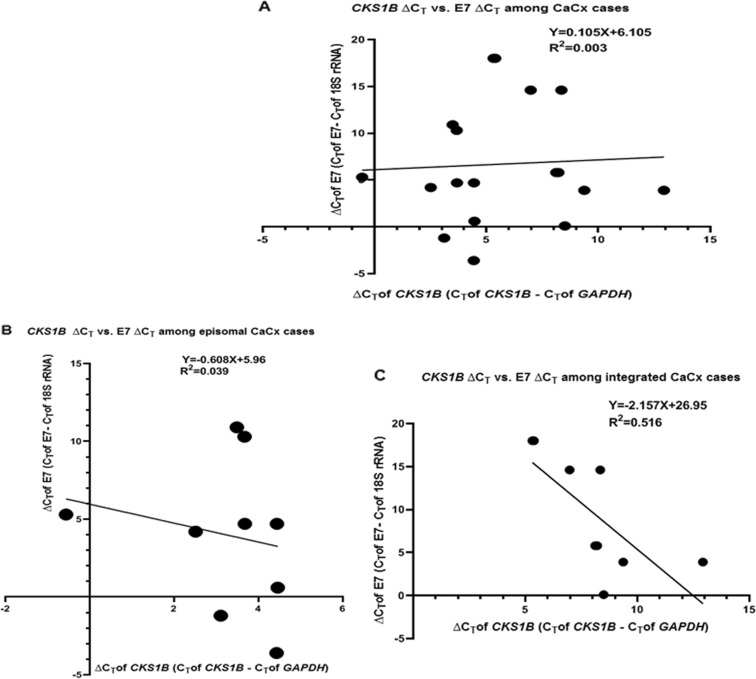
Correlation between CKS1B and E7 expression among cervical cancers. Linear regression analysis of the correlation between *CKS1B* mRNA expression and E7 mRNA expression in (**a**) CaCx cases (episomal and integrated; *p* = 0.839), (**b**) episomal CaCx cases (*p* = 0.61), and (**c**) integrated CaCx cases (*p* = 0.03). Lower ∆C_T_ means higher expression

In an earlier study^13^, we recorded that the two categories of CaCx cases differ with respect to levels of viral oncogene E7 expression. Hence, it became necessary to explore if the viral oncogene expression had any influence on the expression of the cellular gene, *CKS1B*, which is targeted by miR-181c. Among all the CaCx cases, irrespective of the viral physical status and among the episomal CaCx cases, no significant correlation was recorded between the expression of these cellular genes and viral oncogene E7. But strikingly, only among integrated CaCx cases, a strong negative correlation was identified between E7 and *CKS1B* expression (*R*^2^ = 0.516; *p* = 0.03) as shown in Fig. 7.

## Discussion

Global gene expression profiling has been used widely to identify genes that are aberrantly expressed in cervical tumours as compared with normal cervix^17,18^, and^19^. Considering our earlier study depicting the relevance of differences between CaCx cases harbouring episomal and integrated viral genomes^13^, in the current study, we chose to identify differentially expressed host genes and relevant pathways for CaCx pathogenesis in general or specifically distinct among episomal and integrated CaCx cases. Besides confirming this, the most important pathways identified for CaCx pathogenesis, irrespective of HPV physical status were the cell cycle regulatory pathways such as “Cell Cycle: G2/M DNA Damage Checkpoint Regulation”, “Cell Cycle Control of Chromosomal Replication, Mitotic Roles of Polo-Like Kinase”, and “Role of BRCA1 in DNA Damage Response”. Altered expression of proteins belonging to these pathways exert direct influence on the efficacy of antitumor agents as well as tumour outcomes^20–23^. Therefore, a clinical correlation of genes captured in the enriched pathways would help in determining the translational efficacy of the findings recorded in this study.

The most significant pathways identified in case of episomal CaCx cases was “CTLA4 signalling in cytotoxic T lymphocytes” and “T-cell receptor signalling”, portraying overexpression of most of the genes captured in such pathways. Such exclusively enriched pathways could probably be attributable to higher viral load and E7 expression, in such cancers harbouring episomal HPV16^13^ and reflective of the role of the tumour immune microenvironment or the role of tumour infiltrating lymphocytes in such CaCx subtypes. Therefore, genes of such pathways could potentially serve as targets for combating such cancers, efficiently. On the other hand, the most significantly enriched pathways in CaCx cases harbouring integrated HPV16 were signalling pathways such as “eIF2 Signalling”, “Regulation of eIF4 and p70S6K Signalling”, and “mTOR Signalling”, together with “Mitochondrial Dysfunction” pathway. Such signalling pathways associate with processes like oxidative stress and DNA repair, autophagy, and cell migration. These observations could be suggestive of the potential role of the host virus fusion proteins that are common among HPV16-integrated CaCx cases, where heterogeneous levels of viral oncoprotein E7 do not correlate with viral copy numbers^13^.

Among the miRNAs with altered expression levels recorded in our study, majority followed expression patterns that have already been recorded in case of CaCx cases, with the exception of miR-200a and miR-181c. The overexpression of miR-200a revealed a pattern that differed from the other miRNAs analysed, suggesting that this miRNA could be associated with HPV16 positivity. A recent study^24^ identified overexpression of miR-200a in HPV induced CaCx and tonsillar cancers, which seems to be consistent with our observation. Another recent study by the Cancer Genome Atlas Research Network, 2017^25^ identified that expression of miR-200 family was negatively correlated with those of the epithelial–mesenchymal transition-related transcription factors. Overexpression of miR-200a in pancreatic cancers^26^ and ovarian cancers^27^ have been recorded and attributed as contributing to the epithelial phenotype and cell proliferation maintaining the tumour bulk, respectively. Although such phenomenon might explain our findings and be prognostically relevant, it could also be worthwhile to hypothesise that this miRNA may serve as an early marker of HPV16 infection, specifically for singling out HPV16-positive women who might be at heightened risk for CaCx development.

Unlike miR-200a, miR-181c showed differentially downregulated expression, exclusively among episomal CaCx cases but not among integrated CaCx cases. miR-181c has already been reported as a p53-miR, which targets a number of components of the miRNA processing complex^28^. A study^29^ suggested that expression level of miR-181c in the serum can be used as a biomarker for therapeutic efficacy in cervical cancers. Thus, miR-181c expression status could potentially serve as a novel risk marker for episomal CaCx cases and calls for functional characterisation of the role of this miRNA in episomal CaCx pathogenesis. We tested this possibility by focussing our attention on the miR-181c target gene, *CKS1B*, which belonged to the significantly enriched pathway in cervical cancers. We identified that *CKS1B* was differentially overexpressed only among episomal CaCx cases, whose expression was significantly and inversely correlated with miR-181c expression in such CaCx subtype.

Enhanced expression of *CKS1B* in a number of cancers such as myeloma, breast cancer, lymphoma, renal carcinoma, ovarian cancer, salivary, and oesophageal cancers etc. has been associated with poor prognosis. This has been attributed to amplification of *CKS1B* gene leading to lymph node metastases^30^. Besides influencing cell growth and survival through regulation of *p27*^*Kip1*^, silencing of *CKS1B* is also known to induce cell death and inhibit growth of tumour cells through mechanisms that are independent of *p27Kip1*and *SKP2*^31,32^. Taken together, our study highlights a novel mechanism of *CKS1B* overexpression in HPV16 episomal CaCx subtypes, which could be attributable to loss of epigenetic regulation of *CKS1B* by miR-181c, which is downregulated in such CaCx subtypes. Thus, this could serve as one of the mechanisms of proliferation activation in this CaCx subtype that could be of translational relevance.

There exist at least two transcript variants of *CKS1B* gene and it appears that only transcript variant 1 encodes the protein^33^. In our study, we employed primers designed to capture the expression of this coding variant of *CKS1B*. Hence, availability of a small molecule inhibitor of *CKS1B*, fluoxetine, which is used as an antidepressant in clinics^34^, suggests that drugging *CKS1B* could be a potential avenue of treating such CaCx subtypes harbouring episomal HPV16, as opposed to those harbouring purely integrated HPV16 and this could be subject to clinical trials.

Our previous study also demonstrated that there exists heterogeneity among integrated CaCx cases, based on viral load and E7 expression level^13^. The negative correlation between E7 expression and expression of *CKS1B* among such cases potentially suggests the complementary role of viral and host proteins, in the pathogenesis of such CaCx cases harbouring integrated HPV16 irrespective of miR-181c (Fig. 7). Thus, our study substantiates that CaCx cases harbouring integrated and episomal HPV16 are distinct at the molecular level.

Besides miR-181c, our study also revealed a strong positive correlation between miR-323 expression and HPV16 E7 expression among episomal CaCx cases. There are reports showing miR-323 upregulation in metastatic squamous cell CaCx cases^35^ and several studies on prostate cancers^36,37^, which are suggestive of the potential of this microRNA in imparting aggressiveness to such cancers. We also validated this through cell line based in vitro analysis as indicated in Fig. 4. This was indicative of the fact that the viral oncogene E7 could regulate host miRNA expression, which calls for further studies on detailed molecular mechanisms. The influence of HPV16 oncoproteins E6 and E7 on miR-203 expression during proliferation and differentiation has already been established^14^. Therefore, the significant positive correlation between the expression of miR-203 and E7 in CaCx subtype harbouring integrated HPV16 also merits attention and further exploration.

In this study, through the correlation analysis between the prioritised set of miRNAs and their target genes (experimentally validated and predicted), we have been instrumental in identifying functional miRNA-mRNA relationships as detailed in Table S6. Infact, a large proportion of the non-validated target genes revealed negative correlations with the respective miRNAs at the level of expression, suggesting that these could be further analysed for exploring the regulation of miRNAs and mRNAs in CaCx pathogenesis.

In summary, employing both microarray-based gene expression profiling and expression analysis of a prioritised set of miRNAs, we have been instrumental in establishing that CaCx cases harbouring both integrated and episomal HPV16 genomes are distinct molecular subtypes. Although the TCGA study on cervical cancers^38^ has been instrumental in highlighting distinct molecular phenotypes of HPV-active and HPV-inactive cancers, we have succeeded in revealing distinct molecular subtypes of HPV16-positive cervical cancers. Our study further demonstrated deregulation of miRNA expression in CaCx cases compared with controls, progressive deregulation of miRNA expression with increase in severity of cervical status based on histopathology and HPV infections, and differential expression of miRNAs in CaCx cases harbouring episomal and integrated viral genomes. Therefore, in consideration with our previous observation of the loss of miRNA-binding sites in viral regulatory regions facilitating viral gene expressions in CaCx cases^39^ and gain of miRNA binding sites in host regulatory regions^40^ we conclude that host cellular miRNAs have a role in CaCx pathogenesis. Thus, our findings highlight that CaCx cases with episomal and integrated HPV16 are distinct entities at the molecular level and such findings are of immense translational value, specifically for designing targeted therapy of CaCx cases.

## Materials and methods

### Samples and subjects

The samples used for this study were nested to an ongoing natural cohort study. The malignant samples selected for the study were histopathologically confirmed invasive squamous cell carcinomas and clinically diagnosed as tumour stage III and above as per FIGO classification. Samples were derived from married subjects attending a cancer referral hospital (Saroj Gupta Centre and Research Institute, South 24 Parganas, West Bengal, India) collected within the period of 1998–2013.

The non-malignant and control samples were derived from married and non-pregnant women with no previous history of cervical dysplasia/malignancy undergone for hysterectomy attending a hospital (Calcutta Medical College Hospital, Kolkata, West Bengal, India). All such samples were collected during the period of 2010–2013, with informed consent approved by the institutional ethical committee for human experimentation. Details regarding DNA isolation, HPV screening and determination of HPV16, physical status of HPV16 genomes, and viral load are described earlier from our laboratory^4,41–44^.

### Microarray-based gene expression analysis

Global gene expression profiling data reported by Sharma et al. (GEO database, NCBI Accession No: GSE67522) was used following reclassification of the CaCx samples based on the physical status of HPV16 genome, to identify mechanistic differences between cervical cancers harbouring episomal and integrated HPV16. Thus, differential expression analysis was performed using different categories of cervical tissues, i.e., HPV-negative control samples (*n* = 11), HPV positive non-malignant samples (*n* = 11), episomal CaCx cases (*n* = 10), and integrated CaCx cases (*n* = 10) (Table S8). The analysis pipeline was the same as reported earlier^15^.

### Expression analysis of some candidate microRNAs

The expression of some prioritised miRNAs (34 miRNAs selected on the basis of frequent alterations in many cancers, identified through literature survey) were determined by TaqMan miRNA assays as well as Power SYBR Green based assays (Supplementary methods 1 and 2 and Figures S7 and S8, respectively) employing 25 HPV-negative normal samples, 25 HPV16-positive non-malignant samples, and 62 HPV16-positive CaCx cases (43 episomal and 19 integrated HPV16 genomes). The various categories of miRNAs analysed, and the assays employed are provided in Supplementary methods 1–3 and Tables S4, Table S9, and Table S10.

### Target prediction of miRNA

The target human genes corresponding to a subset of the miRNAs analysed in the study, were predicted based on three prediction softwares like miRanda, PICTAR, and TargetScan, considering validated miRNA binding sites as well.

### Validation of miRNA target gene CKS1B by quantitative real-time PCR

Validation was done considering only *CKS1B* gene that appeared as target of the significantly altered miR-181c recorded among episomal CaCx cases only. This gene was selected based on the fact it belonged to the significantly enriched pathway corresponding to the CaCx cases. Samples used for validation by quantitative real-time PCR included episomal CaCx cases (*n* = 43), integrated CaCx cases (*n* = 19), HPV-negative controls (*n* = 25), and HPV16-positive non-malignant samples (*n* = 25). The details of primer sequences of *CKS1B* and *GAPDH*, amplicon size and PCR conditions employed is depicted in Table S10.

### Cell culture and transfection

HPV-negative cell line C33A and HPV16-positive cell lines Caski and SiHa were cultured in Dulbecco's Modified Eagle Medium supplemented with 10% fetal bovine serum, 50 Units/ml of penicillin and 50 µg/ml of streptomycin at 37 °C and 5% CO_2_. C33A cells were transfected using Lipofectamine 2000 reagent according to manufacturer’s protocol, using 1 µg of plasmid pcDNA3.1-HPV16 E7 vector generated in our laboratory^15^. The cells were harvested and washed with 1× PBS (pH 7.4), trypsinized, and collected by centrifugation at 300 g for 10 min The transfected cells were used further for RNA isolation. The transfection experiments were carried out in three sets, each in triplicates and selected miRNAs expression was carried out in selected cell lines according to the protocol described earlier.

## Supplementary information

Supplementary data

## References

[1] Das D, Bhattacharjee B, Sen S, Mukhopadhyay I, Sengupta S (2010). Association of viral load with HPV16 positive cervical cancer pathogenesis: Causal relevance in isolates harboring intact viral E2 gene. Virology.

[2] Thomas M, Massimi P, Navarro C, Borg JP, Banks L (2005). The hScrib/Dlg apico-basal control complex is differentially targeted by HPV-16 and HPV-18 E6 proteins. Oncogene.

[3] Romanczuk H, Howley PM (1992). Disruption of either the E1 or the E2 regulatory gene of human papillomavirus type 16 increases viral immortalization capacity. Proc. Natl. Acad. Sci. USA.

[4] Bhattacharjee B, Sengupta S (2006). CpG methylation of HPV 16 LCR at E2 binding site proximal to P97 is associated with cervical cancer in presence of intact E2. Virology.

[5] Umbach JL (2008). MiRNAs expressed by herpes simplex virus1 during latent infection regulate viral mRNAs. Nature.

[6] Skalsky RL, Cullen BR (2010). Viruses, microRNAs, and Host Interactions. Annu. Rev. Microbiol..

[7] Guo YE, Steitz JA (2014). Virus meets host MicroRNA: the destroyer, the booster, the hijacker. Mol. Cell Biol..

[8] Calin GA (2004). Human microRNA genes are frequently located at fragile sites and genomic regions involved in cancers. Proc. Natl. Acad. Sci. USA.

[9] Farazi TA, Hoell JI, Morozov P, Tuschl T (2013). microRNAs in human cancer. Adv. Exp. Med. Biol..

[10] Calin GA, Croce CM (2006). MicroRNA-cancer connection: the beginning of a new tale. Cancer Res..

[11] Sharma G, Dua P, Agarwal SM (2014). A comprehensive review of dysregulated miRNAs involved in cervical cancer. Curr. Genomics.

[12] Jiménez-Wences H (2016). Methylation and expression of miRNAs in precancerous lesions and cervical cancer with HPV16 infection. Oncol. Rep..

[13] Das Ghosh D (2012). Some novel insights on HPV16 related cervical cancer pathogenesis based on analyses of LCRmethylation, viral load, E7 and E2/E4 expressions. PLoS. ONE.

[14] McKenna DJ, McDade SS, Patel D, McCance DJ (2010). MicroRNA 203 expression in keratinocytes is dependent on regulation of p53 levels by E6. J. Virol..

[15] Sharma S (2015). Bridging links between long noncoding RNA HOTAIR and HPV oncoprotein E7 in cervical cancer pathogenesis. Sci. Rep..

[16] Bartel DP (2009). MicroRNAs: target recognition and regulatory functions. Cell.

[17] Ahn WS (2004). Searching for pathogenic functions to cervical cancer. Gynecol. Oncol..

[18] Medina-Martinez I (2014). Impact of gene dosage on gene expression, biological processes and survival in cervical cancer: a genome-wide follow-up study. PLoS. ONE.

[19] Lee HS (2015). Identification of differentially-expressed genes by DNA methylation in cervical cancer. Oncol. Lett..

[20] Takai N, Hamanaka R, Yoshimatsu J, Miyakawa I (2005). Polo-like kinases (Plks) and cancer. Oncogene.

[21] Kauffmann A (2008). High expression of DNA repair pathways is associated with metastasis in melanoma patients. Oncogene.

[22] Chen RH, Tian YJ (2013). Enhanced anti-tumor efficacy of aspirin combined with triptolide in cervical cancer cells. Asian Pac. J. Cancer Prev..

[23] Ujhelyi Z (2015). The enhanced inhibitory effect of different antitumor agents in self-microemulsifying drug delivery systems on human cervical cancer HeLa cells. Molecules.

[24] Vojtechova Z (2016). Comparison of the miRNA profiles in HPV-positive and HPV-negative tonsillar tumors and a model system of human keratinocyte clones. BMC Cancer.

[25] Cancer Genome Atlas Research Network. (2017). Integrated genomic and molecular characterization of cervical cancer. Nature.

[26] Li A (2010). Pancreatic cancers epigenetically silence SIP1 and hypomethylate and overexpress miR-200a/200b in association with elevated circulating miR-200a and miR-200b levels. Cancer Res..

[27] Liu N (2015). Upregulation of microRNA-200a associates with tumor proliferation, CSCs phenotype and chemosensitivity in ovarian cancer. Neoplasma.

[28] Boominathan L (2010). The tumor suppressors p53, p63, and p73 are regulators of MicroRNA processing complex. PLoS. ONE.

[29] Wang WT (2014). Differentially expressed microRNAs in the serum of cervical squamous cell carcinoma patients before and after surgery. J. Hematol. Oncol..

[30] Zhang Y (2010). CKS1B (CDC28 protein kinase regulatory subunit 1B). Atlas Genet. Cytogenet. Oncol. Haematol..

[31] Liberal V (2012). Cyclin-dependent kinase subunit (Cks) 1 or Cks2 overexpression overrides the DNA damage response barrier triggered by activated oncoproteins. Proc. Natl. Acad. Sci. USA.

[32] Lee SW (2014). Overexpression of CDC28 protein kinase regulatory subunit1B confers an independent prognostic factor in nasopharyngeal carcinoma. APMIS.

[33] Fagerberg L (2014). Analysis of the human tissue-specific expression by genome-wide integration of transcriptomics and antibody-based proteomics. Mol. Cell Proteom..

[34] Chayka O, D'Acunto CW, Middleton O, Arab M, Sala A (2015). Identification and pharmacological inactivation of the MYCN gene network as a therapeutic strategy for neuroblastic tumor cells. J. Biol. Chem..

[35] Ding H, Wu WL, Wang YX, Zhu FF (2014). Characterization of the microRNA expression profile of cervical squamous cell carcinoma metastases. Asian Pac. J. Cancer Prev..

[36] Gao Q, Yao X, Zheng J (2015). MiR-323 inhibits prostate cancer vascularization through adiponectin receptor. Cell Physiol. Biochem..

[37] Gao Q, Zheng J (2018). microRNA-323 upregulation promotes prostate cancer growth and docetaxel resistance by repressing p73. Biomed. Pharmacother..

[38] Banister CE, Liu C, Pirisi L, Creek KE, Buckhaults PJ (2017). Identification and characterization of HPV-independent cervical cancers. Oncotarget.

[39] Mandal P (2013). Differential expression of HPV16 L2 gene in cervical cancers harboring episomal HPV16 genomes: influence of synonymous and non-coding region variations. PLoS. ONE.

[40] Sharma Saha S (2016). Identification of genetic variation in the lncRNA HOTAIR associated with HPV16-related cervical cancer pathogenesis. Cell. Oncol..

[41] Bhattacharjee B, Sengupta S (2006). HPV16 E2 gene disruption and polymorphisms of E2 and LCR: some significant associations with cervical cancer in Indian women. Gynecol. Oncol..

[42] Bhattacharya P, Sengupta S (2007). Predisposition to HPV16/18-related cervical cancer because of proline homozygosity at codon 72 of p53 among Indian women is influenced by HLA-B*07 and homozygosity of HLA-DQB1*03. Tissue Antigens.

[43] Laikangbam P (2007). A comparative profile of the prevalence and age distribution of human papillomavirus type 16/18 infections among three states of India with focus on northeast India. Int. J. Gynecol. Cancer.

[44] Bhattacharjee B, Mandal NR, Roy S, Sengupta S (2008). Characterisation of sequence variations within HPV16 isolates among Indian women: prediction of causal role of rare non-synonymous variations within intact isolates in cervical cancer pathogenesis. Virology.

