# Supplementary material for: Cervical cancer subtypes harbouring integrated and/or episomal HPV16 portray distinct molecular phenotypes based on transcriptome profiling of mRNAs and miRNAs
Source: Cell Death Discov. 2019 Mar 25;5:81. doi: 10.1038/s41420-019-0154-x (PMC6433907; doi:10.1038/s41420-019-0154-x)
Supplement: Supplementary file 1 — Supplementary data [file 41420_2019_154_MOESM1_ESM.doc]

**Supplementary data**

**Supplementary Methods:**

**1. Relative quantification of mature microRNAs by TaqMan based real time PCR**

Total RNAs, from the cervical tissue samples were isolated, purified and treated with DNase I using the Qiagen RNeasy kit following the manufacturer's protocol. TaqMan miRNA Assays for the selected miRNAs were undertaken, employing cDNA prepared from total RNA samples, using specific miRNA primers from the TaqMan miRNA Assays and reagents from TaqMan miRNA Reverse Transcription Kit (ABI; Cat#4366596). The 15 µl reverse transcription reactions consisted of 100 ng of total RNA, 5 U MultiScribe Reverse Transcriptase, 0.5 mM of each dNTP, 1X reverse transcription buffer, 4 U RNase inhibitor, and nuclease-free water. The reaction was performed at 16°C for 30 min and at 42°C for 30 min, terminated at 85°C for 5 min. For real-time PCR of TaqMan miRNA Assays, we used 0.5 µl 20X TaqMan miRNA Assay Primer, 1.33 µl undiluted cDNA, 5 µl 2XTaqMan Universal PCR Master Mix and 3.17 µl nuclease-free water. The real time PCR program included initial denaturation at 95°C for 10 minutes, followed by 40 cycles of denaturation at 95°C for 15 seconds and annealing at 60°C for 1 minute. The PCR-controls were NTC (non-template control) as well as separate aliquots from Reverse Transcription reactions with (i) all reagents except mRNA, (ii) mRNA and all reagents but no Reverse Transcriptase. Each assay was performed at least thrice, with three replicates per sample in each assay, on MicroAmp optical 96-well plates using a 7900 HT PCR System (ABI). Relative expression of the miRNAs were calculated using RNU6b (TaqMan miRNA control assay) as the endogenous control, and calibrated to the control samples. Representative amplification plots are depicted in **Figure S6**.

**2. Relative quantification of mature microRNAs by Power SYBR Green based real time PCR**

Total RNAs, from the cervical tissue samples were isolated, purified and treated with DNase I using the Qiagen RNeasy kit following the manufacturer's protocol. Power SYBR Green based miRNA Assays for the selected miRNAs were undertaken, employing cDNA prepared from total RNA samples, using specific miRNA stem loop primers and reagents from TaqMan miRNA Reverse Transcription Kit (ABI; Cat#4366596). The 15 ml reverse transcription reactions consisted of 100 ng of total RNA, 5 U MultiScribe Reverse Transcriptase, 0.5 mM of each dNTP, 1X reverse transcription buffer, 4 U RNase inhibitor, and nuclease-free water. This was performed at 16°C for 30 min and at 42°C for 60 min, followed by termination at 85°C for 2 min. For real-time PCR of Power SYBR Green based miRNA assays, the reaction mix was comprised of 0.5 µl miRNA Assay Primer, 0.5 µl universal reverse Primer (stem loop specific), 1.5 µl undiluted cDNA, 5 µl Power SYBR Green PCR Master Mix (Applied Biosystems) and 2.5 µl nuclease-free water. The real time PCR program included initial denaturation at 95°C for 10 minutes, followed by 40 cycles of denaturation at 95°C for 15 seconds and annealing at 60°C for 1 minute. Dissociation curve analysis was done, in order to rule out the occurrence of non-specific amplification and primer dimer formation. The PCR-controls were NTC (non-template control) as well as separate aliquots from Reverse Transcription reactions with (i) all reagents except mRNA, (ii) mRNA and all reagents but no Reverse Transcriptase. . Each assay was performed at least thrice, with three replicates per sample in each assay, on MicroAmp optical 96-well plates using a 7900 HT PCR System (ABI). Relative expressions of the miRNAs were calculated using miR-127 (miRNA control assay which showed uniform expression across all samples) as the endogenous control, and calibrated to the control samples. Representative amplification plots and dissociation curves are depicted in **Figure S7**.

**3. Relative quantification of target genes of miR-181c by Power SYBR Green based real time PCR**

SYBR GREEN based quantitative real time PCR was performed, using the same set of samples used for miRNA expression analysis. For this assay, 100 ng of cDNA was used in a 10 µl reaction mixture with Power SYBR Green PCR Master Mix (Applied Biosystems) and 25 ng of both forward and reverse primers. *GAPDH* expression was also quantified by real time PCR, in a reaction volume of 10 µl including 100 ng of cDNA and 25 ng of forward and reverse primers. *GAPDH* expression served as the internal control, to ensure the integrity of the total RNA sample. Dissociation curve analysis was done, in order to rule out the occurrence of non-specific amplification and primer dimer formation. The PCR-controls were NTC (non-template control) as well as separate aliquots from Reverse Transcription reactions with (i) all reagents except mRNA, (ii) mRNA and all reagents but no Reverse Transcriptase.

The sequences of all primers used in this study are provided in **Table S9.**

**Supplementary figures:**


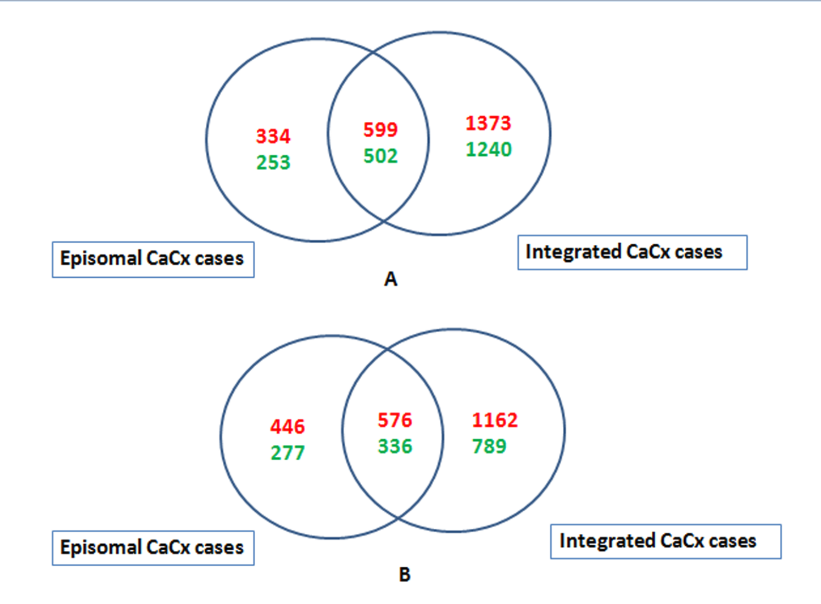


**Figure S1: Differential gene expression among the** **categories of cervical samples. A) Venn diagram represents differentially expressed genes among the two categories of CaCx cases (episomal and integrated) compared to HPV negative controls. B) Venn diagram represents differentially expressed genes among the two categories of CaCx cases (episomal and integrated) compared to HPV16 positive non malignant samples.** **Red represents upregulation and green represents downregulation.**


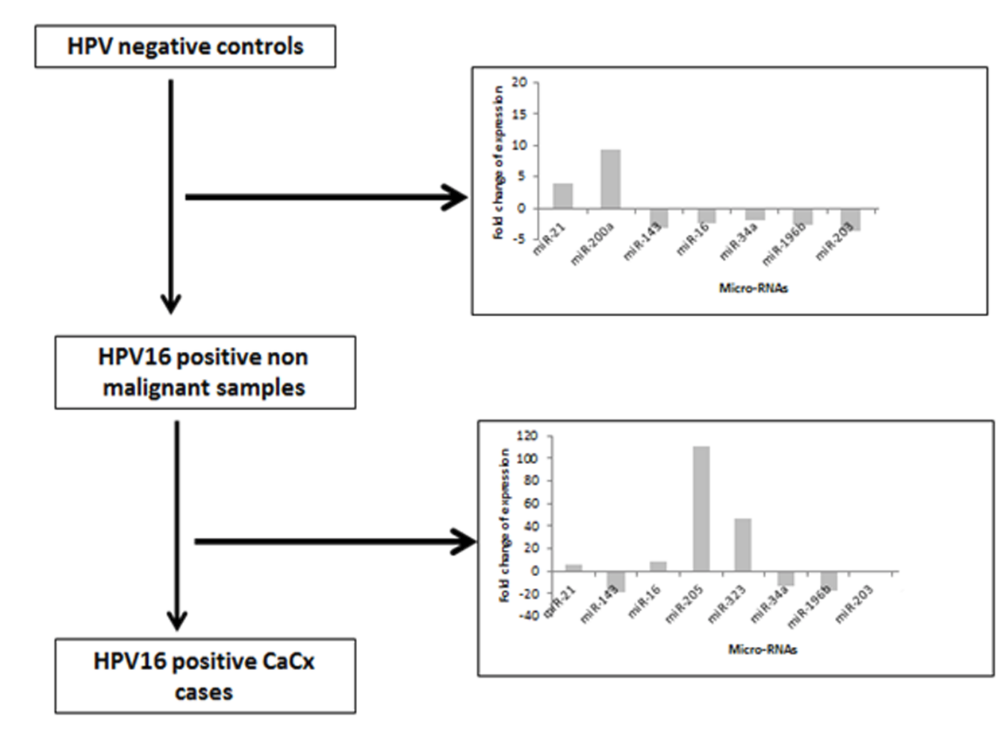


**Figure S2: Diagrammatic representation of trend of miRNAs expression among CaCx cases and HPV16 positive non malignant samples compared to HPV negative controls.**

*
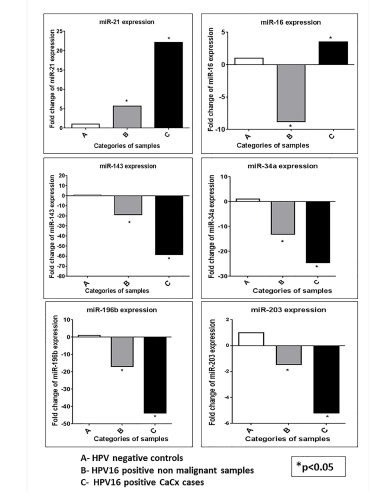
*

**Figure S3: Trends of progressive upregulation (miR-21 and miR-16) and downregulation (miR-143, miR-34a, miR-196b and miR-203) of miRNA expression among CaCx cases and HPV16 positive non-malignant samples, compared to HPV negative controls.**


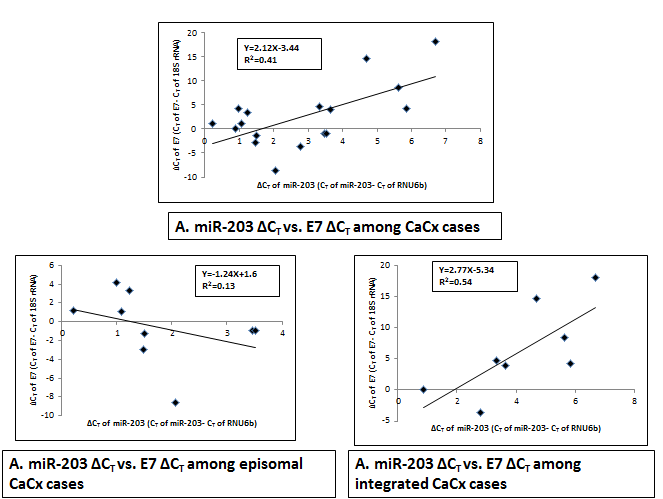


**Figure S4: Linear regression analysis of the correlation between miR-203 expression normalized with RNU6b expression (miR-203 C_T_ – RNU6b C_T_) and E7 mRNA expression normalized with 18S rRNA expression (E7 C_T_ - 18S rRNA C_T_) in A. CaCx cases (episomal and integrated) (p=0.006), B. Episomal CaCx cases) (p=0.334) and C. Integrated CaCx cases (p=0.037).** *Lower ∆C_T_ means higher expression.*


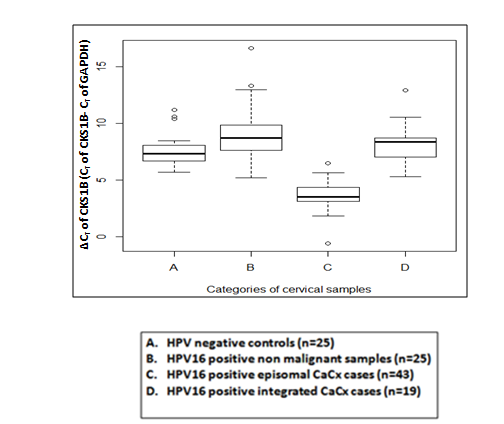


**Figure S5:** Box plots representing distribution of *CKS1B* expression (normalized with endogenous control *GAPDH*) among different categories of cervical samples. *Lower ∆C_T_ means higher expression.*


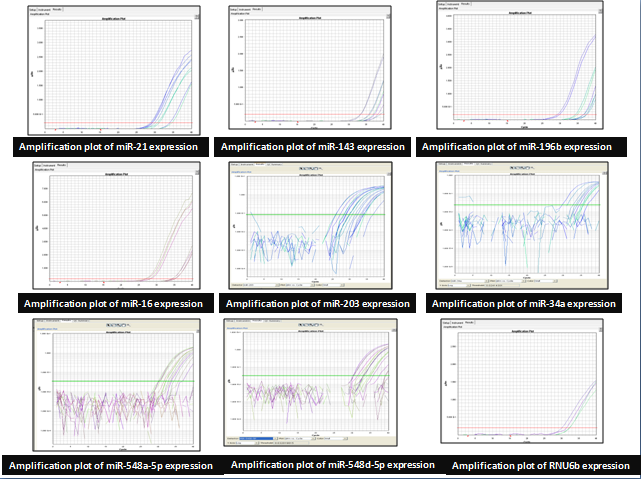


**Figure S6.** Representative amplification plot of candidate miRNAs (miR-21, miR-143, miR-196b, miR-16, miR-203, miR-34a, miR-548a-5p, miR-548d-5p) expression and endogenous control miRNA (RNU6b) expression

**
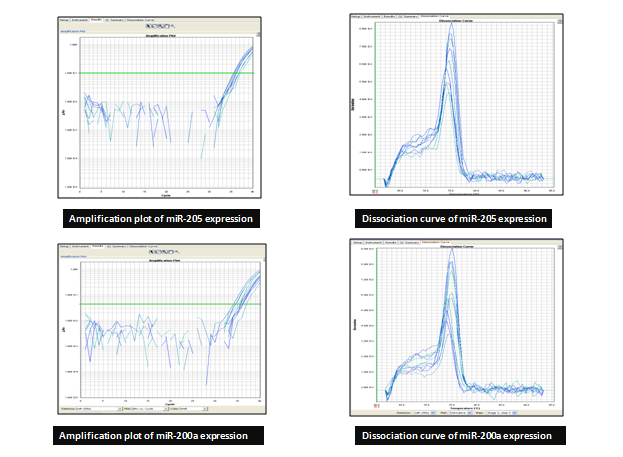

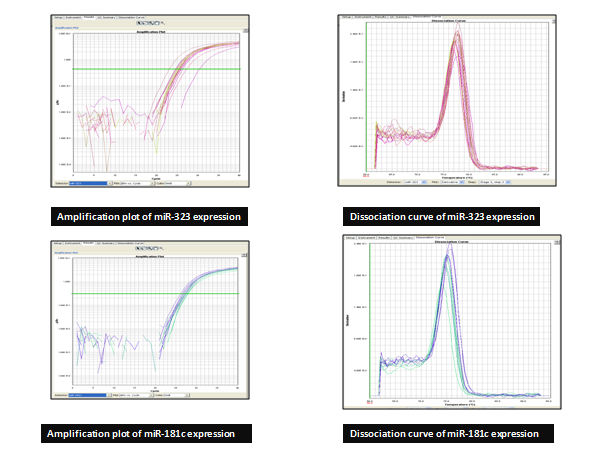
**

**Figure S7.** Representative amplification plot and dissociation curve of some candidate miRNAs (miR-205, miR-200a, miR-323, miR-181c) expression.

**Supplementary tables:**

**Table S1: The top biological processes identified by IPA among CaCx cases (episomal and integrated)**

| **Categories of samples** | **Biological processes** | **p value** | **No. of genes** |
| --- | --- | --- | --- |
| CaCx cases (episomal and integrated HPV16) | Uterine serous papillary cancer | 2.62E-30 | 72 |
|  | Endometrial cancer | 3.02E-26 | 86 |
|  | Mitosis | 6.85E-26 | 97 |
|  | Cell cycle progression | 1.41E-24 | 165 |
|  | Uterine cancer | 1.59E-23 | 130 |
| CaCx cases with episomal HPV16 | Proliferation of cells | 7.53E-07 | 171 |
|  | Infection by Retroviridae | 3.88E-06 | 50 |
|  | HIV infection | 4.32E-06 | 49 |
|  | Quantity of IgG | 2.10E-05 | 20 |
|  | Viral Infection | 4.17E-05 | 89 |
| CaCx cases with integrated HPV16 | Expression of RNA | 2.66E-12 | 348 |
|  | Transcription of RNA | 2.05E-11 | 308 |
|  | Proliferation of cells | 3.87E-11 | 535 |
|  | Proliferation of tumor cell lines | 1.97E-09 | 245 |
|  | Metabolism of protein | 3.07E-08 | 150 |

**Table S2: Pathway analysis of differentially expressed genes in CaCx cases (episomal and integrated HPV16)**

| **Categories of samples** | **Ingenuity canonical pathways** | **P value** | **Total no. of genes captured** | **Upregulated genes** | **Downregulated genes** |
| --- | --- | --- | --- | --- | --- |
| CaCx cases (episomal and integrated HPV16) | Cell Cycle: G2/M DNA Damage Checkpoint Regulation | 5.01E-12 | 17 | *BRCA1, CHEK1, CHEK2, PLK1, MYT1, CDC2, CCNB2, CKS1B, TOP2A, RPS6KA1, CDKN2A, CDC25B, CDC25C, CKS2* | *RPRM, KAT2B, KITLG* |
|  | Cell Cycle Control of Chromosomal Replication | 7.41E-10 | 13 | *ORC1L, ORC6L, CDC6, MCM3, MCM4, MCM5, MCM6, MCM7, MCM8, CDC45L, CHEK2, CDK2, RPA3* | NONE |
|  | Mitotic Roles of Polo-Like Kinase | 5.37E-07 | 12 | *CDC2, PLK1, CCNB2, MYT1, CHEK2, CDC25B, KIF11, HSP90AA1, PRC1, CDC20, PTTG1, KIF23* | NONE |
|  | Role of *BRCA1* in DNA Damage Response | 6.46E-07 | 14 | *FANCC, FANCG, FANCD2, BRCA1, RFC4, RFC5, PLK1, STAT1, CHEK1, CHEK2, SMARCC2, SMARCA4, ACTL6A* | *SMARCD3* |
| CaCx cases with episomal HPV16 | CTLA4 signalling in cytotoxic T lymphocytes | 0.0015 | 7 | *FYN, LCK, CD3E, ZAP70, CD8A, PTPN22* | *PPP2R5A* |
|  | T cell receptor signalling | 0.0006 | 10 | *FYN, LCK, PTPN7, CD3E, ZAP70, NFKB2, CD8A* | *MAPK3, MAP3K1, RASA1* |
| CaCx cases with integrated HPV16 | EIF2 Signaling | 5.01E-13 | 43 | *EIF5, EIF2AK1, PIK3R3, EIF2B5, EIF2AK2* | *RPL22, RPL27A, RPS11, RPS3A, EIF4G2, MAPK3, RPL19, RPL36, RPL12, RPL3, RPLP0, RPL10A, EIF3M, RPS6, RPS4X, RPSA, RPS18, RPL26, RPS17, EIF4E, RPS9, EIF3A, RPS3, RPS5, RPL31, RPL4, RPL37A, PIK3C2A, RPS2, RPL17, RPL30, RPL21, RPL9, EIF3F, RPL32, EIF2B1, EIF2AK3, RPS14* |
|  | Regulation of eIF4 and p70S6K Signaling | 1.17E-06 | 24 | *ITGA3, PPM1J, PIK3R3, EIF2B5* | *RPS18, RPS17, RPS11, EIF4E, RPS3A, EIF4G2, MAPK3, RPS9, EIF3A, RPS3, RPS5, PIK3C2A, RPS2, EIF3M, RPS6, RPS4X, EIF3F, EIF2B1, RPS14, RPSA* |
|  | Mitochondrial Dysfunction | 2.19E-05 | 25 | *SDHB, ATP5D, COX10, CPT1B, RHOT2, PDHA1, SOD2, NDUFS2, COX4I1, NDUFS8, BACE2, TXNRD2* | *PRDX5, ATP5G2, CYB5R3, ATP5F1, COX4I2, ATP5A1, BACE1, COX7A1, NDUFA13, NDUFA6, NDUFB7, CYB5A, CYCS* |
|  | mTOR Signaling | 2.69E-05 | 27 | *RHOT2, VEGFA, PPM1J, PIK3C2A, PIK3R3* | *RPS18, RPS17, PRKAG1, RPS11, EIF4E, RPS3A, EIF4G2, RHOT1, MAPK3, RPS9, GPLD1, EIF3A, RPS5, RPS3, RPS2, RHOJ, EIF3M, RPS6, RPS4X, EIF3F, RPS14, RPSA* |

**Table S3: Upstream regulation for alteration of differential gene expression among CaCx cases**

| **Upstream regulators of CaCx cases (episomal and integrated)** | **Upstream regulators of episomal CaCx cases** | **Upstream regulators of integrated CaCx cases** |
| --- | --- | --- |
| *CDKN1A* (inhibited) | TCR | *MYCN* (inhibited) |
| *TP53* (inhibited) | CD3 | Sirolimus (activated) |
| *E2F1* (activated) |  |  |

**Table S4**.Categories of miRNAs selected for the study

| **Category** | **Micro RNAs**  **Analyzed** |
| --- | --- |
| Enhance tumour growth and proliferation | miR-21,miR-19a,miR-301,miR-135b,miR-190 ,miR-199a ,miR-199a*,miR-200a,miR-222,miR-132 |
| Differentiation regulatory miRNAs | miR-143,miR-150,miR-299,miR-335 |
| Target tumour suppressors and tumour suppressor miRNAs | miR-126 ,miR-16 ,miR-100 ,miR-135a,miR-205 ,miR-323, miR-214,miR-125b,miR-15b,miR-138 ,miR-145,miR-148a,miR-181c,miR-218,miR-338 ,miR-34a. |
| Metastatic suppressor miRNAs | miR-196b. |
| Mesenchymal to epithelial transition miRNAs | miR-203 |
| Metastatic miRNAs | miR-127,miR-182 |

**Table S5: Expression status of different categories of miRNAs among HPV16 positive non malignant samples compared to HPV negative controls**

| **microRNAs** | **Comparisons** | |
| --- | --- | --- |
|  | **HPV16 positive non malignant samples vs. HPV negative controls** | |
|  | **Mann-Whitney U p-value (fold change)** | **FDR of 0.05** |
| miR-21 | **<0.001** (3.94)  upregulation | 0.0015 |
| miR-200a | **<0.001** (9.45)  upregulation | 0.01 |
| miR-143 | **0.001** (-3.09)  downregulation | 0.006 |
| miR-16 | **<0.001** (-2.5)  downregulation | 0.003 |
| miR-34a | **0.004** (-1.87)  downregulation | 0.012 |
| miR-196b | **0.001** (-2.59)  downregulation | 0.007 |
| miR-203 | **<0.001** (-3.55)  downregulation | 0.004 |

- ***Bold indicates significant p value***

**Table S6: Predicted and va**lidated target genes captured in microarray data

| miRNAs | Expression status in CaCx cases | No. of predicted target genes | No. of predicted target genes captured in microarra-y data | No. of differentially expressed predicted target genes captured in microarray data | No. of differentially expressed predicted target genes captured in microarray data (fold change≥2 or ≤-2) | No. of differentially expressed experimental-ly validated target genes captured in microarray data |
| --- | --- | --- | --- | --- | --- | --- |
| miR-21 | Upregulated | 6618 | 1241 | 6 | 5 | 2 |
| miR-143 | Downregulated | 9789 | 1109 | 22 | 18 | None |
| miR-16 | Upregulated | 18478 | 2923 | 7 | 5 | 1 |
| miR-196b | Downregulated | 7746 | 492 | 11 | 9 | None |
| miR-203 | Downregulated | 10324 | 1835 | 37 | 22 | 2 |
| miR-34a | Downregulated | 10039 | 2223 | 43 | 32 | 5 |
| miR-200a | Upregulated in CaCx cases as well as HPV16 positive non malignant samples | 8096 | 1039 | 1 | 1 | None |
| miR-205 | Upregulated | 8972 | 1232 | 7 | 5 | 1 |
| miR-323 | Upregulated | 6203 | 523 | 9 | 7 | None |
| miR-181c | Downregulated among only episomal CaCx cases | 9305 | 1916 | 65 | 44 | 1 |

**Table S7: Characteristic of the selected target gene**

| **Gene** | **Log Fold Change** | **Adjusted P-value** | **Gene name** | **Pathways involved** |
| --- | --- | --- | --- | --- |
|  | *Episomal CaCx cases | Episomal cases |  |  |
| *CKS1B* | 4.26 | 0.01 | CDC28 protein kinase regulatory subunit 1B | Cell Cycle: G2/M DNA Damage Checkpoint Regulation |

** in comparison to controls*

**Tabl**es S8. Categories of samples selected for the study

| **Category of samples** | **Characteristic features** |
| --- | --- |
| HPV negative control samples | 1. HPV negative  2. Histopathologically non malignant |
| HPV16 non malignant samples | 1. HPV16 positive  2. Histopathologically non malignant |
| Episomal HPV16 positive CaCx cases | 1. HPV16 positive  2. Histopathologically squamous cell carcinoma stage II and above  3. APOT assay confirmed E2/E4 and E7 mRNA expression |
| Integrated HPV16 positive CaCx cases | 1. HPV16 positive  2. Histopathologically squamous cell carcinoma stage II and above  3. APOT assay confirmed E7 mRNA expression but no E2/E4 mRNA expression |

**Table S9**: Sequences of primers used for the study of the miRNAs expression along with the amplification conditions

| **miRNAs** | **Stem loop primers for cDNA synthesis of mature miRNAs**  **(5’-3’)** | **miRNA assay primers for real time PCR**  **(5’-3’)** | **Conditions** |
| --- | --- | --- | --- |
| miR-19a | CTC AAC TGG TGT CGT GGA GTC GGC AAT TCA GTT GAG TCA GTT TT | ***Forward:***  ACA CTC CAG CTG GGT GTG CAA ATC TAT GCA A  ***Reverse:*** Universal reverse primer | Initial denaturation: 95°C / 10m  Number of cycles: **4**0  Denaturation: 95°C / 15s  Annealing: 60°C / 1m  Dissociation stage: 95°C/15s, 60°C/15s, 95°C/15s |
| miR-205 | CTC AAC TGG TGT CGT GGA GTC GGC AAT TCA GTT GAG CAG ACT CC | ***Forward:***  ACA CTC CAG CTG GGT CCT TCA TTC CAC CGG  ***Reverse:*** Universal reverse primer | -do- |
| miR-301 | CTC AAC TGG TGT CGT GGA GTC GGC AAT TCA GTT GAG GCT TTG AC | ***Forward:***  ACA CTC CAG CTG GGC AGT GCA ATA GTA TTG T  ***Reverse:*** Universal reverse primer | -do- |
| miR-135b | CTC AAC TGG TGT CGT GGA GTC GGC AAT TCA GTT GAG CAC ATA GG | ***Forward:***  ACA CTC CAG CTG GGT ATG GCT TTT CAT TCC  ***Reverse:*** Universal reverse primer | -do- |
| miR-190 | CTC AAC TGG TGT CGT GGA GTC GGC AAT TCA GTT GAG ACC TAA TA | ***Forward:***  ACA CTC CAG CTG GGT GAT ATG TTT GAT ATA  ***Reverse:*** Universal reverse primer | -do- |
| miR-199a | CTC AAC TGG TGT CGT GGA GTC GGC AAT TCA GTT GAG GAA CAG GT | ***Forward:***  ACA CTC CAG CTG GGC CCA GTG TTC AGA CTA C  ***Reverse:*** Universal reverse primer | -do- |
| miR-199a* | CTC AAC TGG TGT CGT GGA GTC GGC AAT TCA GTT GAG AAC CAA TG | ***Forward:***  ACA CTC CAG CTG GGT ACA GTA GTC TGC ACA  ***Reverse:*** Universal reverse primer | -do- |
| miR-200a | CTC AAC TGG TGT CGT GGA GTC GGC AAT TCA GTT GAG ACA TCG TT | ***Forward:***  ACA CTC CAG CTG GGT AAC ACT GTC TGG TAA  ***Reverse:*** Universal reverse primer | -do- |
| miR-222 | CTC AAC TGG TGT CGT GGA GTC GGC AAT TCA GTT GAG GAG ACC CA | ***Forward:***  ACA CTC CAG CTG GGA GCT ACA TCT GGC TAC TG  ***Reverse:*** Universal reverse primer | -do- |
| miR-126 | CTC AAC TGG TGT CGT GGA GTC GGC AAT TCA GTT GAG GCA TTA TT | ***Forward:***  ACA CTC CAG CTG GGT CGT ACC GTG AGT AA  ***Reverse:*** Universal reverse primer | -do- |
| miR-132 | CTC AAC TGG TGT CGT GGA GTC GGC AAT TCA GTT GAG CGA CCA TG | ***Forward:***  ACA CTC CAG CTG GGT AAC AGT CTA CAG CCA  ***Reverse:*** Universal reverse primer | -do- |
| miR-150 | CTC AAC TGG TGT CGT GGA GTC GGC AAT TCA GTT GAG CAC TGG TA | ***Forward:***  ACA CTC CAG CTG GGT CTC CCA ACC CTT GTA  ***Reverse:*** Universal reverse primer | -do- |
| miR-299 | CTC AAC TGG TGT CGT GGA GTC GGC AAT TCA GTT GAG ATG TAT GT | ***Forward:***  ACA CTC CAG CTG GGT GGT TTA CCG TCC CAC  ***Reverse:*** Universal reverse primer | -do- |
| miR-335 | CTC AAC TGG TGT CGT GGA GTC GGC AAT TCA GTT GAG ACA TTT TT | ***Forward:***  ACA CTC CAG CTG GGT CAA GAG CAA TAA CGA A  ***Reverse:*** Universal reverse primer | -do- |
| miR-100 | CTC AAC TGG TGT CGT GGA GTC GGC AAT TCA GTT GAG CAC AAG TT | ***Forward:***  ACA CTC CAG CTG GGA ACC CGT AGA TCC GAA  ***Reverse:*** Universal reverse primer | -do- |
| miR-135a | CTC AAC TGG TGT CGT GGA GTC GGC AAT TCA GTT GAG TCA CAT AG | ***Forward:***  ACA CTC CAG CTG GGT ATG GCT TTT TAT TCC T  ***Reverse:*** Universal reverse primer | -do- |
| miR-323 | CTC AAC TGG TGT CGT GGA GTC GGC AAT TCA GTT GAG AGA GGT CG | ***Forward:***  ACA CTC CAG CTG GGG CAC ATT ACA CGG TCG  ***Reverse:*** Universal reverse primer | -do- |
| miR-214 | CTC AAC TGG TGT CGT GGA GTC GGC AAT TCA GTT GAG CTG CCT GT | ***Forward:***  ACA CTC CAG CTG GGT CGG ATC CGT CTG AGC  ***Reverse:*** Universal reverse primer | -do- |
| miR-125b | CTC AAC TGG TGT CGT GGA GTC GGC AAT TCA GTT GAG TCA CAA GT | ***Forward:***  ACA CTC CAG CTG GGT AGC AGC ACA TCA TGG  ***Reverse:*** Universal reverse primer | -do- |
| miR-15b | CTC AAC TGG TGT CGT GGA GTC GGC AAT TCA GTT GAG TGT AAA CC | ***Forward:***  ACA CTC CAG CTG GGT AGC AGC ACA TCA TGG  ***Reverse:*** Universal reverse primer | -do- |
| miR-138 | CTC AAC TGG TGT CGT GGA GTC GGC AAT TCA GTT GAG GAT TCA CA | ***Forward:***  ACA CTC CAG CTG GGA GCT GGT GTT G  ***Reverse:*** Universal reverse primer | -do- |
| miR-145 | CTC AAC TGG TGT CGT GGA GTC GGC AAT TCA GTT GAG AAG GGA TT | ***Forward:***  ACA CTC CAG CTG GGG TCC AGT TTT CCC AGG AA  ***Reverse:*** Universal reverse primer | -do- |
| miR-148a | CTC AAC TGG TGT CGT GGA GTC GGC AAT TCA GTT GAG ACA AAG TT | ***Forward:***  ACA CTC CAG CTG GGT CAG TGC ACT ACA GAA  ***Reverse:*** Universal reverse primer | -do- |
| miR-181c | CTC AAC TGG TGT CGT GGA GTC GGC AAT TCA GTT GAG ACT CAC CG | ***Forward:***  ACA CTC CAG CTG GGA ACA TTC AAC CTG TCG  ***Reverse:*** Universal reverse primer | -do- |
| miR-218 | CTC AAC TGG TGT CGT GGA GTC GGC AAT TCA GTT GAG ACA TGG TT | ***Forward:***  ACA CTC CAG CTG GGT TGT GCT TGA TCT AA  ***Reverse:*** Universal reverse primer | -do- |
| miR-338 | CTC AAC TGG TGT CGT GGA GTC GGC AAT TCA GTT GAG TCA ACA AA | ***Forward:***  ACA CTC CAG CTG GGT CCA GCA TCA GTG ATT T  ***Reverse:*** Universal reverse primer | -do- |
| miR-127 | CTC AAC TGG TGT CGT GGA GTC GGC AAT TCA GTT GAG AGC CAA GC | ***Forward:***  ACA CTC CAG CTG GGT CGG ATC CGT CTG AGC  ***Reverse:*** Universal reverse primer | -do- |
| miR-182 | CTC AAC TGG TGT CGT GGA GTC GGC AAT TCA GTT GAG TGT GAG TT | ***Forward:***  ACA CTC CAG CTG GGT TTG GCA ATG GTA GAA  ***Reverse:*** Universal reverse primer | -do- |

**Universal reverse primer**- 5’- TGG TGT GGT GGA GTC G-3’

**Table S10**: Sequences of primers used for the study of the miRNA target genes along with the amplification conditions

| **Experiment** | **Primers**  **(5’-3’)** | **Gene** | **Amplicon (bp)** | **Conditions** |
| --- | --- | --- | --- | --- |
| miRNA (miR-181c) target genes expression | ***Forward:***  TGA TCC ATG AAC CAG AAC C  ***Reverse:***  CAA AGT GAG AAA CAA GAA GG | *CKS1B* | 170 bp | Initial denaturation: 95°C / 10m  Number of cycles: 40  Denaturation: 95°C / 15s  Annealing: 60°C / 1m  Dissociation stage: 95°C/15s, 60°C/15s, 95°C/15s |
|  | ***Forward:***  CAG CCT CAA GAT CAT CAG CA  ***Reverse:***  TGT GGT CAT GAG TCC TTC CA | *GAPDH* | 106 bp |  |
